# Eyes Wide Shut: Primary Process Opens Up

**DOI:** 10.3389/fpsyg.2020.00145

**Published:** 2020-03-03

**Authors:** Justine Bruxelmane, Jonathan Shin, Giulia Olyff, Ariane Bazan

**Affiliations:** ^1^Faculté des Sciences Psychologique et de l’Education, Université libre de Bruxelles (ULB), Brussels, Belgium; ^2^Faculté des Sciences Psychologique et de l’Education, Centre de Recherche en Psychologie Clinique, Psychopathologie et Psychosomatique, Université libre de Bruxelles (ULB), Brussels, Belgium

**Keywords:** eyes, primary process, alpha, language, Freud, association

## Abstract

Freud was the first to invite his patients to lie down on a couch, facilitating the closing of the eyes. If the mere fact of closing the eyes favors access to unconscious materials, it should also favor primary process mentation. Primary process is an associative mode of thought based on superficial similarities including phonology, while secondary process mentation in language is primarily concerned with meaning. Fifty-two participants were given French Word Lists with phonological choices (P) corresponding to primary processes, while semantic choices (S) represent secondary processes when they are in mutual competition (PS). For example, participants were given a first word, such as, e.g. *cale* (to hold), and then had to choose between *lac* (P; lake) and *fixe* (S; fix), which alternative was most similar to the first word, *cale*. Two control lists, SN and PN, where the other choice is unrelated (N for nothing), verify that the subjects are equally capable of recognizing the phonological and semantics similarity. Results show an (near 10% increase in P choices in PS when participants close their eyes, while results on PN and SN were unchanged. The mere fact of closing the eyes induces a modest increase in primary process mentation. Based on the literature, the eyes closed (EC) condition is linked to increased alpha synchronization, which is thought to induce an inward mental shift. This research contributes to validating the psychoanalytic technique consisting on inviting patients to lie down on a couch and invite them to close their eyes.

## Introduction

### Primary and Secondary Processes

The primary and secondary processes are two mental dynamics proposed by Freud (1895/1950) in the first pages of the *Project for a Scientific Psychology*. They constitute the two axes that hold the skeleton of the mental architecture. The priority of the primary processes is to evacuate neuronal excitations as quickly and easily as possible. Primary process dynamics resolve the tensions, among others those caused by the drives and are therefore at the service of the pleasure principle (Freud, 1905/1960). Mentally, the primary processes underlie an associative mode of thought based on superficial similarities – even if these associations turn out to be illogical or contradictory, or even absurd (Freud, 1905/1960). The Hungarian psychoanalyst Rapaport (1951, p. 708) summarizes this as follows: “Where the primary process… hold[s] sway… everything belongs with everything that shares an attribute of it.” Secondary processes are fundamentally heterogeneous with respect to primary processes. By mechanisms of inhibition and selection, they control the excitation discharge to be guided by an intention and not by similarity: these excitations then are said “bound” (Freud, 1895/1950, p. 425).

Mentally, the secondary processes take the context into consideration, enabling the organism to respond more adequately to the drives. Secondary processes thus function according to the reality principle (Freud, 1900/1950) and are said to be rational. It is important to emphasize that thought processes are not primary or secondary in the absolute, but always relative to one another. Both are inevitably linked and function “concurrently and jointly” (Green, 1995, p. 52).

### Primary and Secondary Processes in Language

Freud (1900/1950, p. 596), in *The Interpretation of Dreams*, repeatedly shows how linguistic stimuli are treated in a primary process mode in dreams, indicating: “In particular, we find associations based on homonyms and verbal similarities treated as equal in value to the rest.” Thus, primary process associations can be phonological in nature, and, at an unconscious level, representations are thought to be associated on the basis of common phonemic attributes. Secondary processes, in contrast, allow rational connections between representations (Freud, 1900/1950) as well as an inhibition of associative thoughts, specific to primary processes (Bazan, 2006, 2007). Moreover, they operate according to the thought identity principle (Freud, 1900/1950), and thus, representations are considered according to their intentionality. When it comes to language, the secondary process thus considers the purpose of the act of speech, which thereby promotes the semantic message of words. Consequently, in language, the central principle of the secondary process is semantics, the meaning of the words.

Throughout his oeuvre, Freud presents many examples of clinical symptoms, characterized by a primary process type of logic, associating semantically distinct words, through phonemes or common parts of words. One of the best-known examples refers to a salient symptom in the “Rat Man” (Freud, 1909/1950). This man, suffering from what now would be considered an obsessive neurosis, fears that his father and his fiancée would be submitted to a torture involving a rat. During his childhood, the Rat Man had a nanny, *Frau Hofrat*, who allowed him to have sexual liberties with her. In addition, his father had long hesitated between two women before getting married: one woman he loved and another chosen by his family. The word “to marry” is *heiraten* in German. During the period of his analysis with Freud, the Rat Man fell prey to a similar choice between a marriage of love and one of reason. Finally, the phoneme group “rat” is also found in *Spielratte*, a gambling debt contracted by his father. In other words, during the course of his analysis, Freud observed the constancy of the phoneme group “rat” in the patient’s history, not on the semantic level but on the phonological level. Indeed, the only consistency between these various existential sections of the patient’s life is the presence of the “rat” phoneme (e.g. Frau Hof*rat*, hei*rat*ten, Spiel*rat*te). This, then, is the central phoneme group reappearing in the Rat Man’s symptom, being haunted with a cruel torture involving a rat.

This type of associations, on the primary process mode, is also illustrated by Bazan (2007, p. 20), in her book *Des fantômes dans la voix*. For example, an English-speaking patient dreams that she is sitting in front of her therapist and that their feet are touching. It is by telling her dream: “We were sitting sole to sole,” that she hears the homophony with “we sat soul to soul,” and she understands the double meaning: their souls touched each other. Lacan (1957), in his “return to Freud,” has systematized these clinical observations and proposed the concept of “the signifier” as a phonological fragment representing a signified, that which is referred to by the signifier. Often the signifier is understood as an acoustic element (e.g. Juignet, 2003); however, as Bazan (2007) showed, this is fundamentally flawed and all psycholinguistic evidence unmistakably show that language is about decoding a phonological gesture, i.e. a motor fragment (e.g. Liberman and Mattingly, 1985; Rizzolatti and Arbib, 1998; Studdert-Kennedy, 2000).

In addition to these clinical observations, experimental studies in psychoanalysis have also considered phonological linguistic processing as evidence for the primary process (Shevrin and Luborsky, 1961; Shevrin and Fisher, 1967; Klein Villa et al., 2006; Steinig et al., 2017; Bazan et al., 2019). In the so-called “rebus studies,” psychoanalysts Shevrin and Luborsky (1961) presented images, such as an image of a tie and one of a knee for very short time intervals (e.g. 6 ms) by means of a tachistoscope. Participants then had to choose words from a list that included among other options the rebus solution, here, “tiny.” The results showed that the subjects, even without seeing the images consciously, were more attracted to words phonologically related to the names of the images shown (e.g. “title” for “tie” or “penny” for “knee”), which is an indication of primary process functioning.

In 1967, Shevrin and the American psychoanalyst Fisher again subliminally presented images forming a rebus – here, the images of a pen and of a knee – but this time before the participants fell asleep. Subjects awakened in a rapid eye movement (REM) sleep phase made more phonological associations based on the subliminal stimuli (e.g. a*ny*, *nei*ther, o*pen*), but there were also more associations on the signifier “penny,” resulting from the rebus condensation between the image names “pen” and “knee.” These processes of metonymic displacement and condensation show mental functioning in the primary process mode. On the other hand, subjects awakened after a non-REM (non-paradoxical) sleep phase made more semantic associations – such as ink, paper, leg, foot – indicative of a secondary process mode, with the intention of the speech action and, thus the semantics, predominating. In other words, there is thought to be rebus resolution during dreams, and this resolution is thought to be in the REM phase, the phase of sleep characterized by dreaming. The psychologist Jana Steinig et al. (2017) replicated this study in German with a rebus composed of images of a comb (*Kamm*) and of a raft (*Floss*) forming the German word *kampflos* (peacefully/without fighting). The results were consistent with the results of the initial study by Shevrin and Fisher (1967).

There is ample research in psycholinguistics (Tanenhaus et al., 1979; McClelland and Rumelhart, 1981; Gernsbacher and Robertson, 1995) suggesting that linguistic processing for understanding – i.e. semantic processing – proceeds by a series of activations and inhibitions. A linguistic stimulus activates a set of associations at several levels (graphemes, phonemes, words, and semantic links), as well as inhibition processes, which, in function of the context, gradually stop the processing of irrelevant graphemes, phonemes, and words, to obtain the final interpretation at each level (McClelland and Rumelhart, 1981). This specifically linguistic treatment of the stimulus material would then correspond to the secondary process. Ariane Bazan, at the Université libre de Bruxelles (ULB) and her students (e.g. Libert, 2014; Michel, 2015; Martin, 2016; Cannova, 2017) have composed lists of triads of French words that probe participants for phonological or semantics similarity choices, for example, the triad *Champ-Hanche/Pré* (in English, field-hip/meadow) in which *Champ* [∫ã]is the prime word and the subject has to choose between *Hanche* [ã∫], the phonological equivalent, and *Pré*, the semantic choice. The idea then is that these lists, respectively, measure primary and secondary process types of linguistic choices.

### Closed Eyes

Although Freud has never explicitly made a connection between the primary process and closed eyes, in *Studies on Hysteria* (Freud and Breuer, 1893-95/1950, p. 107), he describes a method involving subjects to close their eyes and having a state of consciousness, “which may in fact have differed very little from a normal one.” He adds: “I ostensibly dropped hypnosis, and only asked for “concentration”; and I ordered the patient to lie down and deliberately shut up his eyes as a means of achieving “concentration.” […] I myself was surprised to find that it yielded me the precise results that I needed” (Freud and Breuer, 1893-95/1950, pp. 109–110). Freud, here, connects the closed eyes state with free association. Since primary processes are fundamentally associative (Freud, 1895/1950), any state encouraging free association actually facilitates the primary process.

In *The Interpretation of Dreams*, Freud (1900/1950, p. 84) proposes specific instructions to encourage the primary process with his free association technique: “For the purpose of self-observation with concentrated attention, it is advantageous that the patient occupy a restful position and close his eyes; he must be explicitly commanded to resign the critique of the thought-formations which he perceives. He must be told further that the success of the psychoanalysis depends upon his noticing and telling everything that passes through his mind, and that he must not allow himself to suppress one idea because it seems to him unimportant or irrelevant to the subject, or another because it seems non-sensical.” The elimination of criticism in this description corresponds to the suspension of the secondary process. In the passage that follows, the link between the resting position, the closed eyes, and the emergence of primary processes is even more salient: “As may be seen, the point is to bring about a psychic state to some extent analogous as regards the apportionment of psychic energy (transferable attention) to the state prior to falling asleep (and indeed also to the hypnotic state)” (Freud, 1900/1950, p. 85).

### Hypothesis

Based on these different elements, the following theoretical hypothesis is proposed: the simple fact of closing the eyes induces a shift of the mental functioning toward a greater mobilization of primary processes. This hypothesis is operationalized thanks to the “Word List tool” (see section “Methodology”), which gives the following operational hypothesis: All things being equal, more phonological targets in the phonologic–semantic triad will be chosen with the eyes closed than with open eyes.

## Methodology

### Population

Version 3.1 of the *G^∗^Power* software (Faul et al., 2009) calculated *a priori* that 54 participants would be required to measure an effect of medium size (effect size *f* = 0.25), for an error α of 5% and a power of 95%. For counterbalancing issues (see section “Counterbalancing and Randomization”), this figure has been reduced to the final *N* of 52 participants.

Demographic data are summarized in Table 1. The average age of participants, aged 18–28, does not differ significantly between men and women [*t*(50) = 1.76, *p* = 0.19]. Forty-nine participants were not studying and had never studied psychology at university. Most subjects were multilingual, with English and/or Dutch being the most frequent secondary languages. Regarding the level of education, the 98% of our sample already had at least a university degree.

**TABLE 1:** Main demographic data (*N* = 52).

Age	% Female	% Polyglots	% College	% Master
21.9 ± 0.3	50	83	63	35

Age in years ± standard error of the mean (SEM). Participants are considered polyglots if they speaks two or more languages; percentage of the participants with a college or master degree are indicated; rest to hundred percent is Bachelor degree.

This study was approved by an ethics committee (ULB), and all participants gave their informed consent in writing.

### Materials

The Word List tool (unpublished) consists of 72 word triads, presented orally. Each triad is composed by a word prime and two target words; the similarity of the targets to the prime is semantic (S), phonological (P), or neutral (N). The semantic similarity corresponds to a non-associative similarity in meaning (e.g. *Cale–Fixe*, in English Hold-Fix). Indeed, associative semantic similarity (e.g. Day–Night) is primary process driven. The phonological similarity is represented here by phonological inverses (e.g. *Rive–Vire*, in English Bank–Turn) and not by rhymes so that the resemblance is not too obvious. The Word List is thus divided into three lists of 24 triads: PS, PN, and SN. The PN and SN lists are control lists that make it possible to verify that the subjects are equally capable of recognizing the phonological and semantics associations in all conditions and that any changes in proportion are not to be interpreted in terms of a variation in competence. Incidentally, as triads are distributed randomly, they also make the logic of PS triads less predictable. Thus, if the lists PN and SN do not report changes, any proportional changes in the choices P or S in PS with closed as opposed to open eyes is to be interpreted in terms of preference in one condition with respect to the other.

### Counterbalancing and Randomization

The 3 lists (24 PS, 24 PN, and 24 SN) are randomly divided into two equal sublists of 12 triads for each participant. The Word List task is an intrasubject task: each participant answers half of the list, i.e. 36 triads, in the control condition (eye open, EO) and the other half in the experimental condition (eye closed, EC). The order of the PS, PN, and SN triads within a condition (EO or EC) is also randomized. The order of the targets in PS (PS and SP), PN (PN and NP), and SN (SN and NS) is counterbalanced by participant. Twenty-six participants first received the EO condition, 26 first the EC condition.

A double-constrained algorithm allowed to equalize the different types of triads (original and inverted) through the 26 versions, which allows each of the 72 triads to appear the same number of times in its original (PS, PN, SN) and in its reversed (SP, NP, NS) form through different versions, and in each of the conditions. Thus, 52 versions of the Word List tool have been created.

In the Word List task, the independent variable is the state of the eyes (open or closed), while the dependent variables are the number of phonological choices in the triads (1) PS and (2) PN and (3) the number of semantic choices in SN triads.

### Procedure

The experiments took place between February and March 2018 in the research laboratory of the Centre de Recherche en Psychology Clinique, Psychopathologie et Psychosomatique at the ULB. The sessions were individual, lasting between 30 and 50 min. During the task, the participant faces an empty wall (∼3 m in front of him), while the experimenter is ∼2 m to his left and out of his field of vision. The chairs have a comfortable but rigid backrest without arm support: the idea is to prevent participants (and the researcher) to relax too much during the tasks.

The participant was asked to complete the anamnestic questionnaire. At the beginning of the experiment, one of the 52 versions was assigned to the subject, which also determined the order of the conditions, either “ECEO” (version V1–V26) or “EOEC” (V27–V52). In versions V1–V26, subjects started with the experimental condition (EC). The researcher began by reading the instruction: “I invite you to close your eyes and keep them closed for a few minutes. I will read words in groups of three: first a one word, then two others. Please choose from the last two words, which choice to you seems most similar to the first word. We will do some examples to start.” He then presented orally three so-called familiarization triads, which were the same for all participants:

*SOURIS* (mouse)//*LAMPE* (lamp)*- MAISON* (house)
*LIVRE* (book)//*NUAGE* (cloud)*- POIVRE* (pepper)
*MEUBLE* (furniture)//*BALLON* (ball)*- SANTÉ* (health)

There was deliberately no phonological or semantic similarity in these triads, the goal being simply to ensure the subject’s understanding of the task. In a second step, the researcher read the Word Lists and surrounded the answer given on a paper version. After the 36th triad, the subject was invited to open his/her eyes for the other half of the task. The instruction then was: “Now, I invite you to open your eyes and keep them open for the rest of the task. This second part is, for the rest, identical to the first. Please, tell me when you’re ready.” In V27–V52, the experiment was exactly the same, but the subjects started with the control condition (EO). The experimental setup (posture of the participant, arrangement of the setup, …) was exactly the same as that described previously, so only the state of the eyes (closed or open) varied between the conditions.

The experiment ended with a debriefing on the Word List to verify a possible awareness of the phonological and semantic links between the primes and target words and a difference between the conditions (EC/EO). Finally, the researcher briefly explained the purpose of the research and answered any questions or comments from participants.

## Results

### Eyes Closed Versus Eyes Open

The average number of phonological (P), semantic (S), and neutral (N) choices are presented in Table 2 (*N* = 52). Participants gave a large majority of semantic responses in PS [μ = 16 ± 5.1; *t*(51) = 5.6, *p* = 0.00] and in SN [μ = 21.2 ± 2.3; *t*(51) = 28.7, *p* = 0.00] and a large majority of phonological responses in PN [μ = 17.5 ± 4.1, *t*(18) = 9.6, *p* = 0.00; out of a total of 24 possible responses for each list]. There is no effect of the order of EO and EC conditions for the S choices in PS [*F*(1.50) = 0.655, *p* = 0.422, bilateral] nor for the S choices in SN [*F*(1.50) = 0.03, *p* = 0.86, bilateral], or for the P choices in PN [*F* (1,50) = 0.07, *p* = 0.79, bilateral].

**TABLE 2:** Mean number of phonological responses in a forced choice between phonological and semantic targets (P/PS), respectively, between P targets and targets bearing no similarity (P/PN) and mean number of S responses in a forced choice between S targets and N targets (S/SN) ± SEM, as well as their proportion on a total of 12 triads (%) when participants close their eyes (EC) as compared to when they open their eyes (EO; *N* = 52).

	P/PS	P/PN	S/SN
EC	4.6 ± 2.7** 38%	8.8 ± 2.3 73%	10.7 ± 1.3 89%
EO	3.4 ± 2.7 29%	8.7 ± 2.4 72%	10.6 ± 1.5 88%

**p < 0.005 for EC as compared to EO.

The intersubject ANOVA analyses also do not indicate any significant difference in function of demographic parameters, be it gender [P/PS *F*(1.50) = 0.10, *p* = 0.75; P/PN *F*(1.50) = 0.36, *p* = 0.55; S/SN *F*(1.50) = 0.28, *p* = 0.60] or education [P/PS *F*(1.50) = 3.25, *p* = 0.08; P PN *F*(1.50) = 0.12, *p* = 0.73; S/SN *F*(1.50) = 0.88, *p* = 0.35]. Note that there is a tendency to choose more P answers in PS for participants with a Master’s degree than for participants with a Bachelor’s degree.

**FIGURE 1:**
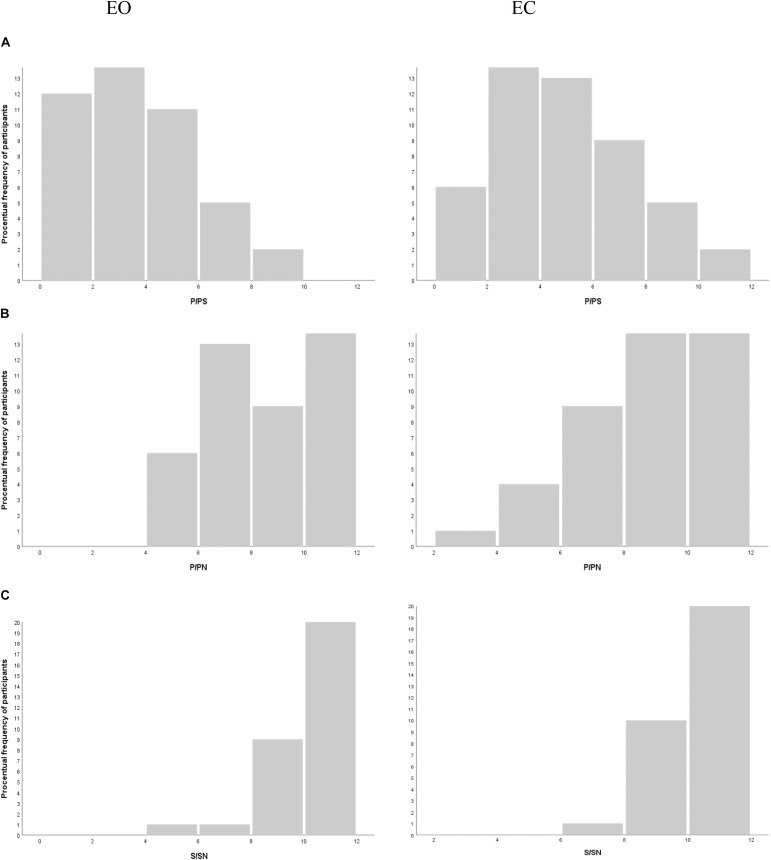
Histograms of the number of **(A)** phonological choices (P) in the PS list, **(B)** P choices in the PN list, and **(C)** semantic choices (S) in the SN for the eyes open (EO) and eyes closed (EC) conditions, respectively.

In the PS triads, the intrasubject ANOVA analysis shows significantly more phonological choices in the experimental EC condition than in the control EO condition [*F*(1,102) = 17.57; *p* < 0.001, bilateral]; this effect size is average (*d* = 0.42) A majority of participants, 65%, make at least one more P choice when they have their EC compared to when their eyes are open. These results are shown in histograms in Figure 1A.

In the PN and SN lists, there was no significant difference in the number of P or S choices, respectively, between the two conditions [P/PN *F*(1,102) = 0.13, *p* = 0.72, bilateral; S/SN *F*(1,102) = 0.17, *p* = 0.68, bilateral]. These results are shown in histograms in Figures 1B,C.

### Debriefing

At debriefing, 60% of the subjects (31 out of 52) reported having felt a difference when their eyes were closed. Of those, only 12 participants (∼23%) claimed it made a difference in the types of targets chosen (phonologically or semantically). However, only five participants (∼10% of the total sample) described it correctly, i.e. they pointed toward a shift toward phonological choices with closed eyes.

## Discussion

The results show an almost 10% increase in phonological choices when the eyes are closed, regardless of the order of presentation and of demographics. Participants were sitting in front of a white wall; the experimenter was sitting a little behind, to their left, outside their field of vision. All were awake and active in both conditions, and were solicited at the same rate, which suggests – even if there is no independent objective measure to confirm this – that all subjects were in the same state of rest (or of wakefulness) under both conditions. We therefore attribute the shift toward P choices simply to the mere fact of closing the eyes. The absence of change in the SN and PN control lists also indicates that this shift is a sign of a preference for phonological choices when the eyes are closed, and is not due to a bad recognition of semantic similarity or to a better recognition of phonological similarity in this condition. Moreover, as the debriefing shows, it seems that this shift toward the phonological is done in an unconscious way, or at least unwittingly, without being able to report it.

These data thus confirm the hypothesis that more phonological choices are made when the eyes are closed than when they are open. This gives credence to our theoretical hypothesis that closing the eyes produces a shift in mental functioning to more primary processes. If the golden rule of the analytic technique is free association (Freud, 1900/1950), it seems justified to encourage participants to close their eyes. However, social conventions make this incitement difficult in a face-to-face conversation. In conclusion, the present results give weight to the technical choice of the analytical treatment to have the analysands lay on a couch, to facilitate the choice to close their eyes and thus facilitate the mental dynamics on the primary process mode that underlies free association.

Paying attention to phonology, and in particular to phonological ambiguity – such as there may exist for example between *rive* and *vire*, *cale* and *lac*, *champ* and *hanche* – is, in fact, a working principle for the clinical psychoanalytic work from a Lacanian point of view. The Lacanian analyst Patrick Gauthier-Lafaye (2017, p. 80) gives us a telling example. He hears an unusual pause in a sentence of a patient: “Ma mère n’était pas parvenue…” (“My mother did not succeed in…”), where the patient pauses in the middle of the word “par-venue” (“succeed”). This slight pause isolates for a suspended moment the embedded phrase “papa revenue,” “daddy has come back.” The analyst simply repeats “pas par’venue,” opening up a new world of meanings. It appeared that the patient’s father left the family without explanation. It had always seemed the minimal duty of the then young woman to be loyal to her mother and to her outrage. Thereby, she could never express her own longings for her father to come back, save for this moment in her analysis 40 years later.

One may of course ask why the mere fact of closing the eyes, regardless of the state of rest, would induce a shift toward an increased primary process mental mode.

### Alpha Waves

There is a brain wave that has been characterized as appearing just by closing one’s eyes and independently of the state of rest: these are alphas waves, neuronal oscillations representing an electrical activity of a frequency between 7.5 and 12.5 Hz (Berger, 1929). The mental state when one closes the eyes is characterized by a maximum amplitude of these alpha waves, and this is called the Berger effect (Berger, 1929). This maximum amplitude of alpha waves when the eyes are closed has been found in many studies (Legewie et al., 1969; Westphal et al., 1993; Barry et al., 2007; Kan et al., 2017), including in children (Barry et al., 2009) and elderly (Barry and De Blasio, 2017). In addition, a recent study by Rimbert et al. (2018) showed an increase in alpha waves in a motor task in the closed-eye condition compared to the open-eye condition. These results confirm that the increase in alpha waves when the eyes are closed is independent of the state of rest.

For alpha synchronization, many empirical studies, done in a conscious state and/or with supraliminal stimuli (Pfurtscheller et al., 1996; Kelly et al., 2006; Klimesch et al., 2007, 2011; Sauseng et al., 2009; Mathewson et al., 2011), have demonstrated its role in shielding against stimuli deemed disruptive by the subject in the given situation. In addition, Wianda and Ross (2019) suggest that alpha synchronization plays an essential role in working memory, as has been demonstrated by several studies (Jensen et al., 2002; Tuladhar et al., 2007; Van Dijk et al., 2010), by inhibiting irrelevant information during memorization. Subjects with closed eyes can be assumed to be in a stronger form of defense against external stimulation, favoring the emergence of internal processes in a primary process mode. In Shevrin and Fisher’s (1967) follow-up studies, Shevrin and Fritzler (1968) and Shevrin et al. (1969, 1970) demonstrate, through evoked potentials (PE) measurements, a link between the resolution of rebuses and alpha waves: the higher the amplitude of the PE response, the more the subjects gave free associations related to the rebus.

Note also that these assumptions are consistent with other observations of the same order. It has been observed, for example, that having the eyes closed promotes the so-called mind wandering, a phenomenon characterized by a detachment of thoughts from the environment (Mason et al., 2007). Smilek et al. (2010, p. 788) propose that closed eyes during mind wandering can reduce or block sensory information and its cortical processing: “the body physically blocks sensory stimulation by reducing exposure of the sensory transducers to external energy sources.” Similarly, several studies (Mason et al., 2007; Christoff et al., 2009) show that closed eyes and mind wandering are strongly linked to the default mode network (DMN). The DMN is a neural network that activates when a subject is not engaged in a task with a specific purpose, during introspective tasks (planning, theory of mind, etc.) or in situations of diffuse attention (Mevel et al., 2010).

All these data converge toward the same hypothesis: the simple fact of closing the eyes would induce an alpha synchronization, which, by shielding against the external stimulation, stimulates the default networks and a shift toward primary associative processes on the basis of internal stimuli; this state would then be comparable to mind wandering. It would seem that Freud, by offering his patients to lie on a couch and invite them to let their thoughts go by closing their eyes, had a genius idea, as suggested by the most recent brain imaging data.

Note, however, that we have not taken any brain measurements, which is a limit to this study: it would seem interesting, or even essential, to verify the neural hypotheses concerning, in particular, alpha synchronization with event-related potential or imaging measurements.

In conclusion, our results show a significant 10% increase in phonological choices, at the expense of semantic choices, when subjects close their eyes. The simple fact of closing the eyes induces a shift toward a more phonological type of language processing that is thought to indicate the implementation of the primary process when the eyes are closed.

## Data Availability Statement

The datasets generated for this study are available on request to the corresponding author.

## Ethics Statement

The studies involving human participants were reviewed and approved by the Ethics Committee of the Université libre de Bruxelles. The patients/participants provided their written informed consent to participate in this study.

## Author Contributions

JB and JS did the bench work and wrote the manuscript. GO and AB supervised the work.

## Conflict of Interest

The authors declare that the research was conducted in the absence of any commercial or financial relationships that could be construed as a potential conflict of interest.
